# RPS3 Aggravates Sepsis-Induced Acute Kidney Injury Through Activating NF-κB Mediated Renal Inflammatory Responses

**DOI:** 10.33549/physiolres.935642

**Published:** 2026-02-01

**Authors:** Xiaoya ZHANG, Qi MA, Jiangzhong WANG, Zhenqi ZHANG, Jun ZHANG

**Affiliations:** 1 Department of Critical Care Medicine, General Hospital of Ningxia Medical University, Yinchuan, Ningxia Hui Autonomous Region, China; 2 School of Computer and Artificial Intelligence, Yinchuan University of Science and Technology, Yinchuan, Ningxia Hui Autonomous Region, China

**Keywords:** Sepsis-associated acute kidney injury, Inflammatory response, Ribosomal protein S3, Nuclear factor-kappa B

## Abstract

Excessive inflammatory responses represent one of the primary causes of sepsis-associated acute kidney injury (S-AKI). The activation of the nuclear factor-kappa B (NF-κB) signaling pathway plays a critical role in the pathogenesis and progression of S-AKI. Previous studies have demonstrated that ribosomal protein S3 (RPS3) serves as a pivotal regulator of the NF-κB pathway; however, its specific biological functions in the context of S-AKI remain to be fully elucidated. This study aims to elucidate the regulatory mechanisms of RPS3 in S-AKI-associated inflammation and to explore the underlying molecular pathways. First, we conducted an analysis of RPS3 level in urine and TNF-α level in serum from S-AKI patients recruited at our hospital. Second, we established a murine model of S-AKI by intraperitoneal injection of LPS, followed by the evaluation of renal function, inflammatory response, RPS3 expression, and NF-κB activation in renal tissues. Finally, we explored the regulatory role and underlying mechanism of RPS3 in the LPS-induced inflammatory response in HK-2 cells through RPS3 knockdown and the introduction of an NF-κB agonist. The results demonstrated that urinary RPS3 and serum TNF-α levels were significantly elevated in patients with S-AKI, with a positive correlation observed between these two parameters. In LPS-induced S-AKI mice, renal function was impaired, accompanied by a robust inflammatory response, increased RPS3 protein expression, and enhanced NF-κB activation in kidney tissue. Knockdown of RPS3 in HK-2 cells mitigated LPS-induced the inflammatory response and suppressed NF-κB activation. However, the effects of RPS3 silencing were partially reversed upon intervention with an NF-κB agonist. Collectively, these findings indicate that RPS3 plays a critical role in the inflammatory response of S-AKI *via* activation of the NF-κB signaling pathway, suggesting its potential as a novel therapeutic target for S-AKI.

## Introduction

Sepsis is one of the leading causes of mortality among patients admitted to the intensive care unit (ICU) [1]. The pathogenesis of sepsis is highly complex. It is not simply an infectious process, but rather an excessive and dysregulated immune response triggered by pathogen invasion. This aberrant response not only fails to effectively clear the pathogen but also leads to widespread host tissue and organ damage, ultimately resulting in multiple organ dysfunction or failure [2]. According to statistical data, approximately 48.9 million individuals are affected by sepsis globally, with a mortality rate reaching as high as 19.7 % [3]. Nevertheless, effective treatments for sepsis remain limited, and the underlying mechanisms of the uncontrolled inflammatory response in sepsis are yet to be fully elucidated.

Sepsis-associated acute kidney injury (S-AKI) is one of the most prevalent and life-threatening complications of sepsis. Unlike isolated acute kidney injury (AKI), the central feature of the pathophysiological process in S-AKI is the inflammatory response induced by sepsis, which results in renal microcirculatory dysfunction as well as metabolic and functional impairment of renal tubular cells. Consequently, S-AKI presents with greater severity compared to either sepsis alone or isolated AKI, and is associated with a significantly higher mortality risk [4]. It is widely acknowledged that the uncontrolled immune-inflammatory response triggered by sepsis can impair renal function, and that inflammatory cytokines may contribute to cellular damage and apoptosis in both glomeruli and renal tubules [5]. Moreover, AKI can also be complicated by sepsis, and the severity of AKI is closely related to the prognosis of sepsis [6]. Currently, the management of S-AKI remains a complex clinical challenge due to the absence of specific pharmacological interventions. Comprehensive treatment strategies are primarily centered on three key areas: infection control, maintenance of systemic homeostasis, and renal function support. However, interventions targeting the first two aspects may inadvertently impose additional stress on the kidneys. Consequently, timely and appropriate initiation of renal replacement therapy has emerged as a critical factor in improving renal outcomes [7,8]. A more in-depth understanding of the underlying pathophysiological mechanisms of S-AKI holds significant potential for identifying novel therapeutic strategies.

The excessive inflammatory cascade is a primary contributor to sepsis-induced kidney injury. Therefore, elucidating the molecular mechanisms underlying inflammation progression may represent a critical focus in the study of S-AKI. Nuclear factor-kappa B (NF-κB) signaling plays a critical role in host defense and is intricately involved in the regulation of both innate and adaptive immune responses [9,10]. Previous research has established that the activation of the NF-κB pathway constitutes a key mechanism underlying the onset and progression of S-AKI [11,12]. Ribosomal protein S3 (RPS3), a constituent of the 40S small ribosomal subunit, exhibits endonuclease activity and is crucial for repairing damaged DNA [13]. In recent years, increasing evidence has highlighted the pivotal role of RPS3 in the inflammatory response [14,15]. Lipopolysaccharide (LPS) constitutes a major component of the outer cell wall in Gram-negative bacteria and is frequently utilized to establish sepsis and S-AKI models [16–18]. Research has demonstrated that the expression of RPS3 in the hippocampus of LPS-induced mice is significantly upregulated, playing a critical role in the neuroinflammatory response associated with sepsis [19]. In addition, RPS3 serves as a critical regulator in the NF-κB signaling pathway, playing an essential role in determining promoter selectivity and transcriptional specificity of NF-κB [20]. Studies have demonstrated that silencing RPS3 can mitigate cigarette smoke-induced acute lung injury by inhibiting NF-κB activity [21]. Therefore, we hypothesize that RPS3 may participate in the immune response through activation of the NF-κB signaling pathway, thereby contributing to the pathogenesis of S-AKI.

In this study, we initially investigated the expression level of RPS3 in S-AKI clinical samples and subsequently elucidated its specific function and underlying molecular mechanisms of RPS3 in S-AKI were investigated through both *in vivo* and *in vitro* experiments. The findings of this study are expected to enrich the understanding of the molecular mechanisms underlying the onset and progression of S-AKI, while also providing novel targets and a robust theoretical foundation for the clinical diagnosis and treatment of S-AKI.

## Materials and Methods

### Clinical samples collection

A total of 30 patients diagnosed with S-AKI who were admitted to the ICU of the General Hospital of Ningxia Medical University between August 2023 and February 2024 were recruited as research participants. The inclusion criteria were as follows: (1) Aged between 25 and 65 years; (2) First-time diagnosis of S-AKI with complete clinical data; (3) Provision of informed consent. The exclusion criteria included: (1) Prior receipt of S-AKI treatment before enrollment; (2) Presence of malignant tumors; (3) Concurrent urinary system diseases; (4) Coexistence of other infections or immune-related disorders. In addition, 14 healthy individuals were recruited to form the healthy control group. Blood and urine samples were obtained from both the control group and the S-AKI group for analysis. This study received approval from the Research Ethics Review Committee of the General Hospital of Ningxia Medical University (KYLL-2022-0637).

### S-AKI model and grouping

Eight-week-old male C57BL/6 mice were obtained from the Laboratory Animal Center of Ningxia Medical University and maintained in a pathogen-free environment with a 12-h light/dark cycle for one week. During this period, the mice had *ad libitum* access to food and water. Sixteen mice were randomly divided into two groups: control and S-AKI (n=8). The mouse model of S-AKI was induced in the S-AKI group by intraperitoneal injection of LPS (200 μl, 10 mg/kg) [22], while the control group received an equivalent volume of normal saline. 24 h post-injection, the mice were anesthetized with sodium pentobarbital (2 %, 45 mg/kg, i.p.), and blood samples were collected *via* orbital puncture. Subsequently, the mice were euthanized by cervical dislocation, and their kidney tissues were promptly harvested. One half of the kidney tissue was fixed in 4 % paraformaldehyde (Sinopharm Chemical Reagent, Shanghai, China), while the other half was stored at −20 °C for further analysis. Animal experiments conducted in this study were approved by the Research Ethics Review Committee of the General Hospital of Ningxia Medical University (KYLL-2022-0637).

### Cell culture and treatment

Human renal proximal convoluted tubule cells (HK-2) were obtained from American Type Culture Collection (ATCC, Rockefeller, USA) and cultured in Keratinocyte Serum-Free Medium (Gibco, Carlsbad, USA). HK-2 cells were exposed to varying concentrations (0, 1, 5, and 10 μg/ml) of LPS (Sigma-Aldrich, St. Louis, USA) for 24 h. The optimal LPS concentration on HK-2 cells was determined based on alterations in cell viability and the expression level of RPS3. Additionally, HK-2 cells were treated with 5 μM of the NF-κB agonist BAY 11-7085 (MedChemExpress, Monmouth Junction, USA) for 3 h [23], followed by collection for further analysis.

### Cell transfection

Three siRNA target sequences were selected based on the transcript sequence of RPS3, followed by the design of three pairs of siRNAs (si-RPS3-1#, sense strand 5′-GGCUGCGAGGUUGUGGUGUCU-3′ and antisense strand 5′-ACACCACAACCUCGCAGCCUU-3′; si-RPS3-2#, sense strand 5′-GAGGAAGUUUGUCGC-UGAUGG-3′ and antisense strand 5′-AUCAGCGAC-AAACUUCCUCUU-3′; si-RPS3-3#, sense strand 5′-CCAGGACAGAAAUCAUUAUCU-3′ and antisense strand 5′-AUAAUGAUUUCUGUCCUGGUU-3′). Subsequently, the corresponding DNA fragments were synthesized *in vitro* and cloned into a vector to generate RPS3 interference plasmids. One day prior to transfection, HK-2 cells were seeded into 6-well plates and cultured until they reached 70 % confluence. The plasmids were then transfected into HK-2 cells using LipofectamineTM 2000 (Invitrogen, Carlsbad, USA). 48 h post-transfection, the expression level of RPS3 in HK-2 cells was assessed to screen the plasmid with the highest interference efficiency.

### Cell counting kit-8 (CCK-8) assay

HK-2 cells were seeded into 96-well plates at a density of 3×10^3^ cells per well and subsequently cultured until adherence was achieved. Following the intervention with LPS and BAY 11-7085, 10 μl of CCK-8 solution (Solarbio, Beijing, China) was added to each well, followed by incubation for 2 h. Finally, the absorbance of the cells at 450 nm was measured using a microplate reader, and the cell viability was calculated accordingly.

### Flow cytometry

HK-2 cells were dissociated using trypsin digestion and subsequently resuspended gently with a pipettor. The resulting cell suspension was transferred to a flow tube. Following centrifugation at 1000 rpm for 5 min, the supernatant was carefully removed. After washing the cells, binding buffer (1×) was added to resuspend the cells to a concentration of 2×10^6^ cells/ml. Then, 100 μl of the cell suspension was carefully aspirated, followed by the addition of 5 μl of Annexin V/FITC and 5 μl of propidium iodide (PI) solution (Solarbio, Beijing, China). The mixture was gently vortexed and incubated at room temperature in the dark for 5 min. Finally, 400 μl of binding buffer (1×) was added to the sample, and flow cytometry analysis was performed.

### Biochemical assay

Serum samples from mice and the culture medium supernatant of HK-2 cells were collected. The levels of proteinuria, blood urea nitrogen (BUN), and creatinine (Cr) in the mouse serum, as well as lactate dehydrogenase (LDH) in the HK-2 cell culture medium, were measured following the instructions provided in the assay kits by Nanjing Jiancheng Bioengineering Institute (Nanjing, China).

### Enzyme-linked immunosorbent assay (ELISA)

Human urine, serum, mouse serum, and HK-2 cell were collected. The concentrations of RPS3, TNF-α, and IL-6 in the samples were quantified following the instructions provided in the assay kits by Jingmei Biotechnology Co., Ltd (Yancheng, China).

### Hematoxylin and eosin (HE) staining

Mice kidney tissues were fixed in 4 % paraformaldehyde for 24 h, embedded in paraffin, sectioned into 4 μm thick slices, deparaffinized with xylene, and hydrated through a graded ethanol series. The sections were stained with hematoxylin for 5 min and eosin (Solarbio, Beijing, China) for 1 min. Following dehydration with ethanol and transparency (Sinopharm Chemical Reagent, Shanghai, China) with xylene, the sections were mounted using neutral gum. Renal histomorphological changes were examined under a microscope, and pathological scores were evaluated [24].

### TdT-mediated dUTP nick-end labeling (TUNEL) staining

After deparaffinization, the mouse kidney tissue sections were treated with proteinase K at 37 °C for 20 min. Subsequently, the membrane-breaking solution was added and incubated at room temperature for 20 min. The TUNEL reaction mixture (Servicebio, Wuhan, China) was prepared according to the manufacturer’s instructions, applied to the tissue sections, and incubated at 37 °C for 1 h. Following this step, the tissues were stained with DAPI solution (Sigma-Aldrich, St. Louis, USA) in the dark at room temperature for 10 min. Finally, following the sealing of the sections with an anti-fluorescence quenching mounting solution, the degree of apoptosis in renal tissue was assessed under a fluorescence microscope.

### Real-time quantitative PCR (RT-qPCR)

Mice kidney tissues and HK-2 cells were harvested, and total RNA was extracted from the samples using TRIpure total RNA extraction reagent (ELK Bio-technology, Denver, USA). Subsequently, first-strand cDNA was synthesized. This cDNA was utilized as a template for RT-qPCR amplification, which was carried out using SYBR Green PCR SuperMix (ELK Biotech-nology, Denver, USA) with the following reaction program: 95 °C for 3 min, followed by 40 cycles consisting of 95 °C for 10 s, 58 °C for 30 s, and 72 °C for 30 s. Finally, the mRNA expression levels of the target genes were quantified using the 2^−ΔΔCt^ method. The primer sequences employed in this experiment are presented in Table 1.

### Western blot analysis

Mice kidney tissues and HK-2 cells were harvested and lysed using RIPA buffer (Solarbio, Beijing, China) supplemented with protease inhibitors for protein extraction. Protein concentrations were quantified using the BCA assay. Subsequently, protein samples were denatured by heat treatment and resolved *via* gel electrophoresis. Proteins were then transferred onto polyvinylidene fluoride (PVDF) membranes (Millipore, Billerica, USA) and blocked with a blocking solution for 2 h at room temperature. The membranes were incubated overnight at 4 °C with primary antibodies after being placed in incubation cartridges. This was followed by incubation with horseradish peroxidase conjugated secondary antibody (1:10000) for 1 h at room temperature. The chemiluminescence reagent was applied to the membrane for development, followed by analysis of the optical density of the protein band to determine the expression level of the target protein. The primary antibodies utilized in this experiment are listed as follows: anti-RPS3 antibody (1:2000), anti-NF-κB p65 antibody (1:1000), anti-NF-κB p65 (phosphor S536) antibody (1:1000), anti-TNF-α antibody (1:2000), anti-IL-6 antibody (1:1000), and anti-GAPDH antibody (1:5000). All the antibodies mentioned above were procured from Abcam (Cambridge, UK).

### Statistical analysis

Statistical analysis was conducted using SPSS (version 22.0) and GraphPad Prism (version 8.4.3). An unpaired *t*-test was employed for comparisons between two groups, while one-way ANOVA followed by Tukey’s *post hoc* test was utilized for comparisons among multiple groups. The correlation between urinary RPS3 levels and serum TNF-α levels in patients with S-AKI was evaluated using SPSS software. A multi-variate linear regression analysis was performed to adjust for the potential confounding effects of patient age, gender, S-AKI severity, and survival status on the association between RSP3 and TNF-α. A *p*-value of less than 0.05 was considered indicative of statistical significance.

## Results

### Urinary RPS3 is increased in patients with S-AKI

**Fig. 1:**
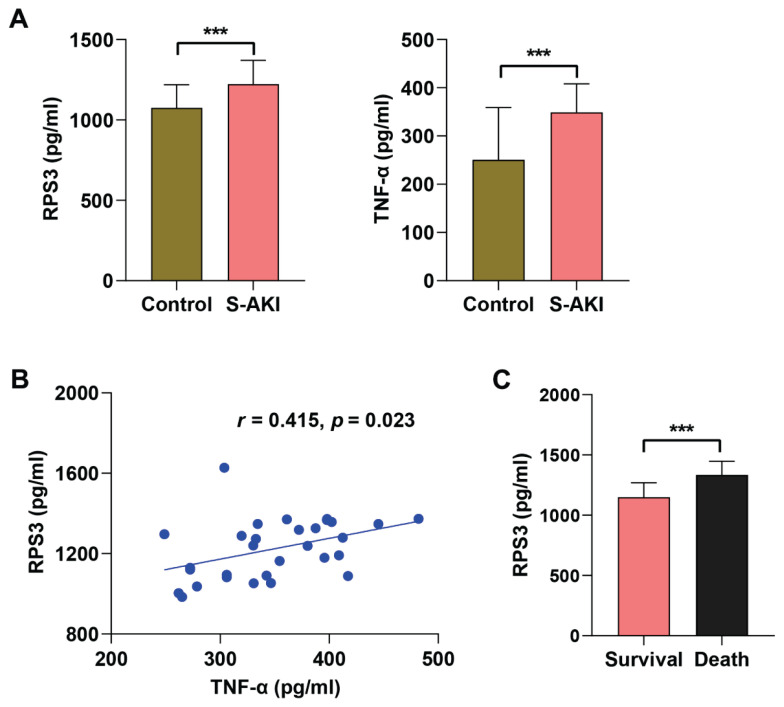
Urinary RPS3 level is abnormal in patients with S-AKI. Urine and blood samples were collected from S-AKI patients (S-AKI group, n=30) and healthy people (Control group, n=14). (**A**) ELISA was employed to quantify the levels of RPS3 in urine and TNF-α in serum. (**B**) Correlation analysis of urine RPS3 level and serum TNF-α level in patients with S-AKI. (**C**) Analysis of RPS3 level in urine of patients with S-AKI who died within 28 days of treatment and those who survived. *** *p*<0.001.

Through the analysis of clinical samples, we observed that the urinary RPS3 level and serum TNF-α level in S-AKI patients were significantly elevated compared to healthy people (Fig. 1A). Furthermore, a positive correlation was identified between urinary RPS3 level and serum TNF-α level in S-AKI patients (Fig. 1B). Through multiple linear regression analysis, we observed that the correlation between RPS3 and TNF-α remained significant after adjusting for patient age and gender. However, this correlation became statistically nonsignificant after further controlling for the severity of S-AKI and patient survival status. Within 28 days post-treatment, 12 patients succumbed to their conditions while 18 patients survived. We subsequently conducted an analysis of the differences in urinary RPS3 level between these two groups of patients and observed that the urinary RPS3 level were significantly elevated in the deceased patients compared to those who survived (Fig. 1C). These findings indicate that elevated RPS3 level may play a role in the pathogenesis and progression of S-AKI and hold potential as a diagnostic and prognostic biomarker for S-AKI.

### RPS3 expression is up-regulated in kidney of LPS-induced S-AKI mice

**Fig. 2:**
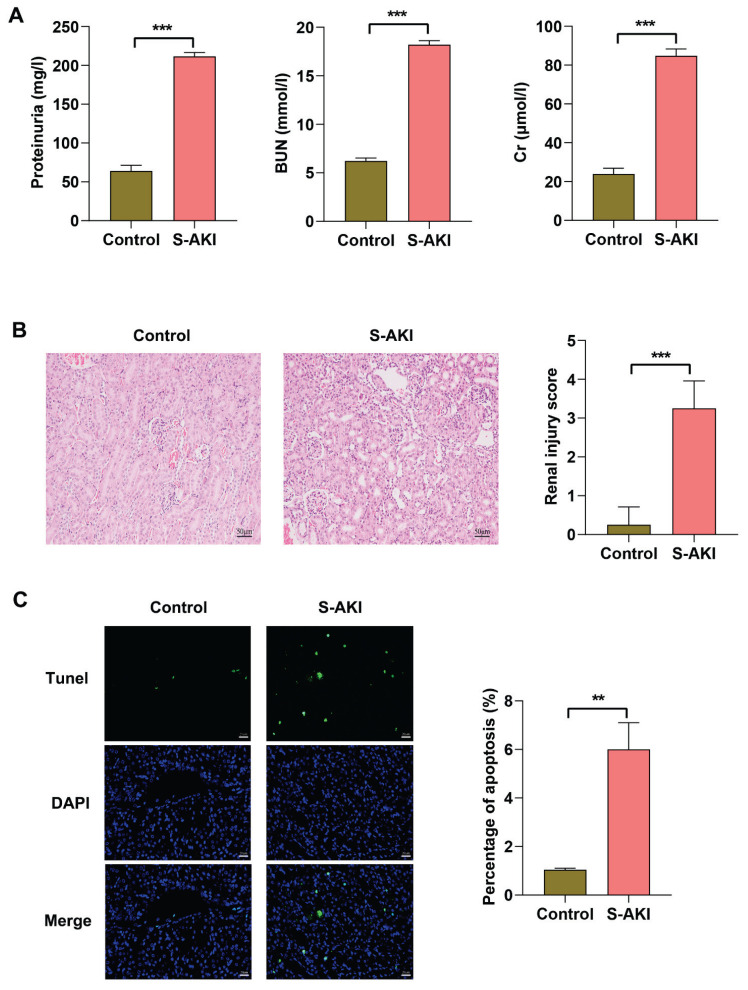
LPS-induced S-AKI mice exhibit pronounced kidney injury. The S-AKI model was established by intraperitoneal injection of 10 mg/kg LPS in C57BL/6 mice and induced for 24 h. (**A**) The levels of proteinuria, BUN, and Cr in serum were measured using biochemical assays. (**B**) The pathological damage of kidney tissue was observed using HE staining, and the injury score was evaluated. Scale bar = 50 μm. (**C**) The level of apoptosis in kidney tissue was assessed using TUNEL staining. Scale bar = 20 μm. ** *p*<0.01, *** *p*<0.001. BUN, urea nitrogen; Cr, creatinine.

**Fig. 3:**
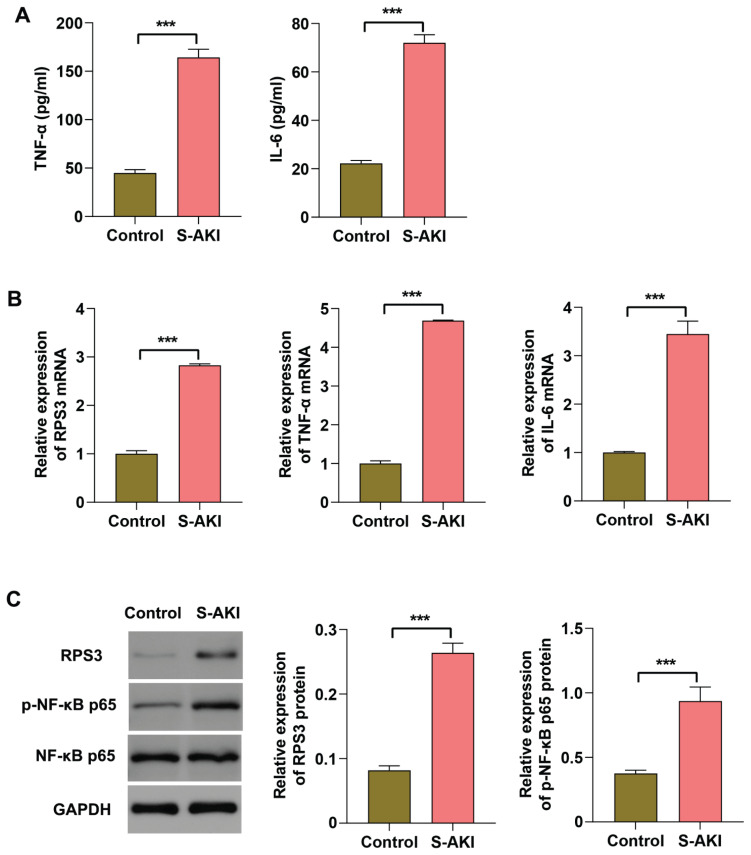
RPS3 expression is up-regulated in kidney of LPS-induced S-AKI mice. The S-AKI model was established by intraperitoneal injection of 10 mg/kg LPS in C57BL/6 mice and induced for 24 h. (**A**) The levels of TNF-α and IL-6 in serum was measured using ELISA. (**B**) The mRNA expression levels of RPS3, TNF-α, and IL-6 in kidney tissue was detected using RT-qPCR. (**C**) The protein expression levels of RPS3 and p-NF-κB p65 in kidney tissue were assessed using Western blot analysis. *** *p*<0.001.

Next, we conducted *in vivo* experiments to further investigate the expression changes of RPS3 in S-AKI animal models. Initially, we employed LPS-induced mice to simulate the pathological condition of S-AKI. Our findings revealed that renal function in these mice was significantly compromised, as evidenced by markedly elevated levels of proteinuria, BUN, and Cr in the serum of S-AKI mice (Fig. 2A). Furthermore, histopathological analyses demonstrated severe damage to the renal tissue of S-AKI mice, characterized by necrosis and sloughing of renal tubular epithelial cells, glomerular ischemia and shrinkage, enlargement of balloon space, infiltration of inflammatory cells, and an increase in apoptotic cells within the renal tissue (Fig. 2B–C). In addition, the contents of TNF-α and IL-6 were significantly elevated in serum of LPS-induced S-AKI mice (Fig. 3A). Notably, the mRNA expression levels of RPS3, TNF-α, and IL-6, as well as the phosphorylation of NF-κB p65 and RPS3 protein, were markedly upregulated in the kidney tissues of S-AKI mice (Fig. 3B–C). In summary, these findings indicate that a pronounced inflammatory response occurs in the kidney tissue of LPS-induced S-AKI mice, which may be associated with the upregulation of RPS3 and the activation of NF-κB p65.

### RPS3 is up-regulated in LPS-induced HK-2 cells

**Fig. 4:**
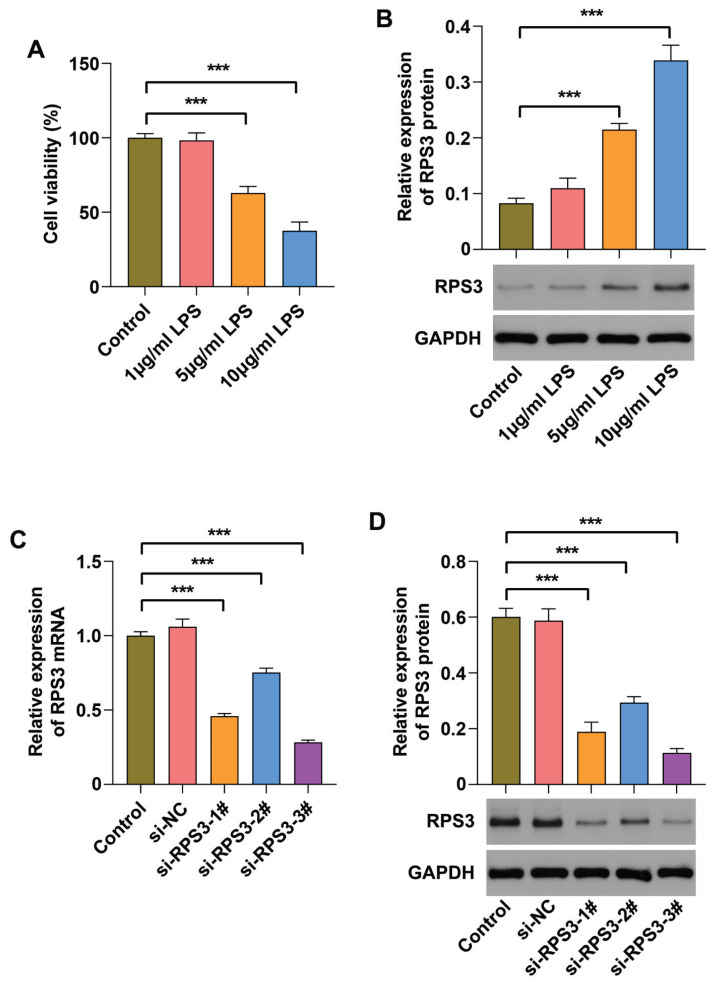
RPS3 is highly expressed in LPS-induced HK-2 cells. HK-2 cells were exposed to varying concentrations (0, 1, 5, and 10 μg/ml) of LPS for 24 h. (**A**) Cell viability was assessed using the CCK-8 assay. (**B**) The protein expression level of RPS3 in HK-2 cells was determined using Western blot analysis. RPS3 interference plasmids were constructed and subsequently transfected into HK-2 cells. (**C~D**) The expression levels of RPS3 mRNA and protein in HK-2 cells were quantified by RT-qPCR and Western blot analysis. *** *p*<0.001.

To further validate the accuracy of the aforementioned findings, LPS-induced HK-2 cells were employed to establish an *in vitro* model simulating S-AKI. It was observed that as the concentration of LPS intervention increased, the viability of HK-2 cells progressively decreased, while the expression level of RPS3 gradually increased (Fig. 4A–B). These results substantiate that the high expression of RPS3 plays a critical role in the onset and progression of S-AKI. Furthermore, we identified the optimal LPS induction concentration of 5 μg/ml, which not only induced HK-2 cell damage but also preserved sufficient cell viability for subsequent experiments, with the expression level of RPS3 being significantly upregulated. Given that RPS3 was upregulated in LPS-induced HK-2 cells, we specifically targeted its expression to investigate its precise role and underlying molecular mechanisms in S-AKI. Consequently, si-RPS3-3#, which exhibited the most efficient interference of RPS3 expression, was selected for subsequent transfection experiments in HK-2 cells (Fig. 4C–D).

### Down-regulation of RPS3 alleviates injury of LPS-induced HK-2 cells by inhibiting NF-κB p65 activation

**Fig. 5:**
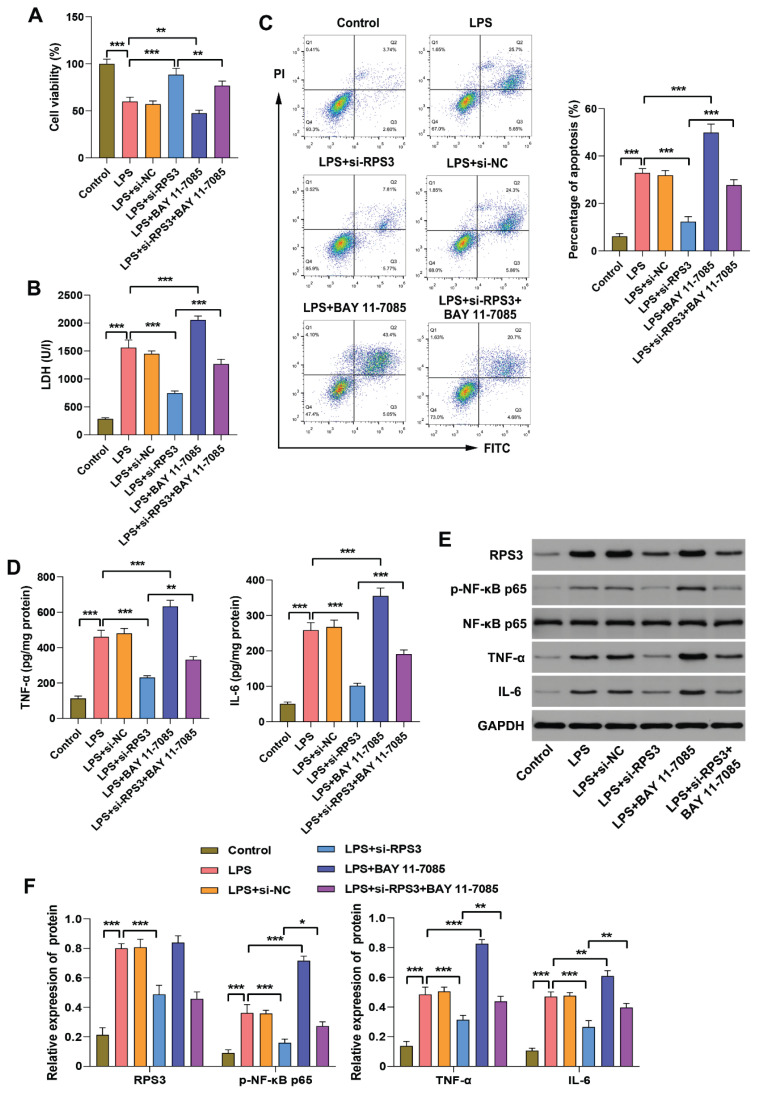
Down-regulation of RPS3 inhibits NF-κB activation and reduces inflammatory response in LPS-induced HK-2 cells. HK-2 cells transfected with si-RPS3 were exposed to 5 μg/ml LPS for 24 h, and the NF-κB agonist BAY 11-7085 was administered 3 h prior to sample collection. (**A**) The viability of HK-2 cells was assessed using the CCK-8 assay. (**B**) The activity of LDH in the supernatant of HK-2 cell culture medium was measured biochemically. (**C**) The apoptosis rate of HK-2 cells was analyzed by flow cytometry. (**D**) The concentrations of TNF-α and IL-6 in HK-2 cell were quantified using ELISA. (**E–F**) The protein expression levels of RPS3, TNF-α, IL-6, and p-NF-κB p65 in HK-2 cells were evaluated using Western blot analysis. * *p*<0.05, ** *p*<0.01, *** *p*<0.001.

Study has demonstrated that RPS3 binds to NF-κB p65, thereby promoting its phosphorylation and activating the NF-κB signaling pathway [25]. In the lung tissues of S-AKI mice, we observed upregulation of RPS3 expression along with increased phosphorylation of NF-κB p65. Therefore, we hypothesized that RPS3 may play a critical role in the inflammatory injury process of S-AKI by mediating the activation of the NF-κB signaling pathway. To investigate the role and underlying molecular mechanisms of RPS3 in the pathophysiology of S-AKI, we conducted *in vitro* experiments by silencing RPS3 expression in HK-2 cells and introducing the NF-κB agonist BAY 11-7085. The results demonstrated that the viability of HK-2 cells was significantly reduced (Fig. 5A), while LDH release and apoptosis were markedly increased upon exposure to LPS (Fig. 5B–C). Following the silencing of RPS3, the viability of LPS-induced HK-2 cells was partially restored, and both LDH release and apoptosis were effectively attenuated. However, intervention with BAY 11-7085 exacerbated LPS-induced cellular damage and apoptosis, and partially counteracted the protective effects of RPS3 down-regulation on damage and apoptosis in LPS-induced HK-2 cells. In addition, LPS stimulation significantly increased the levels of TNF-α and IL-6 (Fig. 5D), as well as the protein expression levels of RPS3, TNF-α, and IL-6, and enhanced the phosphorylation of NF-κB p65 in HK-2 cells (Fig. 5E–F), indicating a robust inflammatory response. Silencing RPS3 effectively attenuated the levels of inflammatory cytokines and suppressed NF-κB acti-vation in LPS-induced HK-2 cells. Conversely, treatment with BAY 11-7085 activated the NF-κB signaling pathway, exacerbating the inflammatory response in LPS-treated HK-2 cells, and partially reversed the inhibitory effects of RPS3 knockdown on inflammatory cytokine production and NF-κB activation. Taken together, these findings indicate that the silencing of RPS3 mitigates inflammatory responses in LPS-induced HK-2 cells through the inhibition of NF-κB activation.

## Discussion

The kidney serves as a critical target organ in the pathophysiological process of sepsis. AKI is a frequent complication in septic patients, characterized by an abrupt decline in renal function and representing a clinical severe disease that affects multiple human body systems. S-AKI has consistently been a research focus in nephrology and critical care medicine. Nevertheless, its intricate pathogenesis poses significant challenges to its clinical management [26]. In this study, we identified that RPS3 was aberrantly overexpressed in S-AKI through clinical sample analysis and *in vivo* animal experiments. Subsequent *in vitro* investigations demonstrated that the downregulation of RPS3 expression could mitigate LPS-induced injury and inflammatory responses in HK-2 cells, potentially *via* the inhibition of NF-κB pathway activation. This study elucidates the biological role and partial molecular mechanisms of RPS3 in S-AKI for the first time, thereby providing a promising therapeutic target for the prevention and treatment of S-AKI.

Early diagnosis and targeted treatment play a crucial role in the prevention and management of S-AKI, which can enhance patient prognosis and decrease healthcare expenditures. Consequently, the significance of biomarkers in diagnosing S-AKI has gained greater attention [27]. Although the KDIGO guidelines utilize urine output and serum creatinine levels as diagnostic criteria for AKI, these indicators are limited in evaluating the extent of kidney injury and also exhibit delays in recognition [28,29]. To date, numerous studies have identified various markers for the early detection of S-AKI, including neutrophil-associated lipid transporter protein (NGAL) [30], kidney injury molecule 1 (KIM-1) [31], TNF-α, Cystatin C [32], and liver-type fatty acid binding protein (L-FABP) [33]. In this study, we observed that the urinary concentration of RPS3 in S-AKI patients was significantly higher than that in healthy individuals, and it was higher in the urine of patients with S-AKI who died. Additionally, a positive correlation was identified between urinary RPS3 levels and serum TNF-α in S-AKI patients. Furthermore, the severity of S-AKI and the survival status of the patients may serve as key contributing factors underlying the observed association between RPS3 and TNF-α. In short, these findings indicate that RPS3 may serve as a potential clinical diagnostic biomarker for S-AKI and could play a role in its pathogenesis.

During the sepsis infection phase, pathogens gain entry into the bloodstream and release toxins, such as LPS, as well as other pathogen-associated molecular patterns (PAMPs). These substances activate immune cells, leading to the excessive production and release of pro-inflammatory cytokines (including TNF-α and IL-6) and chemokines. This cascade results in an uncontrolled and systemic inflammatory response, commonly referred to as a cytokine storm [34]. Mesangial cells within the glomerulus not only secrete substantial amounts of inflammatory mediators but also exhibit structural and functional alterations in response to pathogenic stimuli, which ultimately leads to a reduction in the glomerular filtration rate [35]. Furthermore, inflammatory mediators activate the coagulation system, leading to intravascular thrombosis, and directly damage endothelial cells, inducing cellular swelling and dysfunction, which further aggravates renal microcirculatory disturbances [5,36]. Additionally, these mediators contribute to increased apoptosis and necrosis of renal tubular epithelial cells [37]. Therefore, in both LPS-induced *in vivo* and *in vitro* models, we observed substantial production of pro-inflammatory cytokines, and S-AKI mice exhibited impaired renal function, renal tissue edema, and increased renal cell apoptosis. However, following the interference of RPS3, these pathological manifestations were significantly ameliorated. These findings suggest that targeting RPS3 may play a crucial regulatory role in the immune response associated with S-AKI; however, its underlying molecular mechanisms remain to be fully elucidated.

NF-κB is a pivotal regulator in the modulation of the immune system. Under normal physiological conditions, NF-κB binds to its inhibitor, IκB, and resides in the cytoplasm in an inactive form [38]. However, upon exposure to endogenous inflammatory signals or stimulation with pathogen derivatives such as LPS, NF-κB becomes activated [39]. Lipid A of LPS serves as the critical component for immune recognition. Upon specific recognition by the Toll-like receptor 4 and myeloid differential protein-2 complex on the cell surface, it binds to this receptor complex, leading to the recruitment of the adaptor protein MyD88 and subsequent activation of downstream signaling pathways. This process results in the phosphorylation and degradation of IκB, enabling the release of NF-κB into the nucleus to initiate gene transcription. Ultimately, this cascade triggers a robust immune response [38]. This process is crucial for the host’s resistance to infection; however, excessive NF-κB activation may result in pathological damage, such as sepsis. Studies have demonstrated that S-AKI mice induced by intraperitoneal injection of LPS exhibit severe kidney injury, immune dysfunction, and activation of the NF-κB signaling pathway in renal tissue [40–42]. In this study, we observed that RPS3 expression was significantly upregulated in the renal tissues of S-AKI mice, concomitant with activation of the NF-κB signaling pathway. Therefore, we hypothesize that the molecular mechanism underlying the involvement of RPS3 in the pathophysiological progression of S-AKI may be associated with the activation of the NF-κB signaling pathway.

HK-2 cells exposed to LPS demonstrated a pronounced inflammatory response, suppressed cell proliferation, and enhanced apoptosis [43]. Consistent with these findings, we observed that LPS induction resulted in cellular damage and the secretion of substantial proinflammatory cytokines in HK-2 cells. Moreover, in alignment with our *in vivo* findings, LPS-induced activation of the NF-κB pathway and upregulation of RPS3 expression were also observed in HK-2 cells. Notably, silencing RPS3 expression attenuated inflammatory injury and apoptosis in LPS-induced HK-2 cells and concurrently inhibited the activation of the NF-κB pathway. These results collectively provide robust evidence that RPS3 serves as a proinflammatory factor in S-AKI. Study has demonstrated that RPS3 activates NF-κB pathway through the promotion of IκB protein ubiquitination and degradation [44]. Additionally, an increasing body of research evidence confirms the regulatory function of RPS3 in the NF-κB signaling pathway [45–47]. In the current study, we employed the NF-κB agonist BAY 11-7085 to intervene in LPS-induced HK-2 cells, which resulted in exacerbated cellular inflammatory damage. More significantly, BAY 11-7085 reversed the inhibitory effect of RPS3 knockdown on LPS-induced inflammatory injury and apoptosis in HK-2 cells. These results confirm the accuracy of our previously mentioned hypothesis.

In conclusion, our study is the first to elucidate the regulatory role of RPS3 in LPS-induced S-AKI inflammation through NF-κB activation, both *in vivo* and *in vitro*. Moreover, our findings from clinical samples offer preliminary evidence supporting the potential application of RPS3 in the diagnosis and treatment of S-AKI. Nevertheless, given the limited size of the clinical sample set analyzed, we currently lack sufficient data to definitively establish RPS3 as a diagnostic marker for S-AKI. In future studies, we aim to expand the sample size and perform more comprehensive analyses to provide robust and conclusive evidence.

## Figures and Tables

**Table 1:** Primers for RT-qPCR.

*Primer*	Forward (5′-3′)	Reverse (5′-3′)
*Mouse-RPS3*	GATGGCTACTCTGGAGTTGAAGTC	CCAAGGAGTTTGTAGCGTAGAGAC
*Mouse-RNF-α*	CTACTCCCAGGTTCTCTTCAAGG	CTCCCAGGTATATGGGCTCATAC
*Mouse-IL-6*	CATCCAGTTGCCTTCTTGGG	TCCAGTTTGGTAGCATCCATCA
*Mouse-GAPDH*	TGAAGGGTGGAGCCAAAAG	AGTCTTCTGGGTGGCAGTGAT
*Human-RPS3*	GGCTGAAGATGGCTACTCTGG	GTTTGTAACGCAGAGACTCTGCC
*Human-GAPDH*	CATCATCCCTGCCTCTACTGG	GTGGGTGTCGCTGTTGAAGTC
